# Efficacy and safety of acupuncture and moxibustion combined with the external application of traditional Chinese medicine in the treatment of primary liver cancer: A protocol for systematic review and meta-analysis

**DOI:** 10.1097/MD.0000000000027659

**Published:** 2021-10-29

**Authors:** Song Wang, Zhuang Xiong, Yangyang Liu, Yan Leng, Houbo Deng, Dong Shen, Xiangtong Meng, Tiejun Liu

**Affiliations:** a Changchun University of Chinese Medicine, 1035 Bo Shuo Road, Changchun City, Jilin Province, China; b Department of Hepatology, First Affiliated Hospital to Changchun University of Chinese Medicine, 1478 Gongnong Road, Changchun City, Jilin Province, China; c Endocrinology, First Affiliated Hospital to Changchun University of Chinese Medicine, 1478 Gongnong Road, Changchun City, Jilin Province, China.

**Keywords:** acupuncture, external application of Chinese medicine, meta-analysis, primary liver cancer, protocol, safety, systematic review

## Abstract

**Background:**

Primary liver cancer (PLC) is one of the most common malignant tumors in the world, and its incidence and fatality rate are increasing year by year. Due to the large population base in China, the aging population is severely affected by environmental pollution, eating habits, and unhealthy lifestyles. And many other influences have caused the number of new PLC cases and deaths in China to rank first in the world. Acupuncture combined with external application of Chinese medicine to treat PLC is currently one of the commonly used treatments in China. However, this combined treatment still lacks evidence-based medicine support. Therefore, this systematic review and meta-analysis aims to evaluate the efficacy and safety of acupuncture combined with external application of traditional Chinese medicine in the treatment of PLC.

**Method:**

We will search PubMed, Web of Science, GCBI, Embase, OVID, AMED, Cochrane Library, CNKI, VIP, CBM, and Wanfang databases. As of September 15, 2021, there are no restrictions on search language, publication time, and publication status. We will use the following medical keywords to search, including: “acupuncture”, “external application of traditional Chinese medicine”, and “primary liver cancer”. At the same time, we will manually search all reference lists from relevant systematic reviews to find other eligible studies. We will use the random effects model in REVMAN v5.3 for meta-analysis. The study for acupuncture combined with Chinese herbal medicine in the treatment of PLC was a randomized controlled study. Two researchers will independently review the research selection, data extraction, and research quality assessments. Finally, we will observe the outcome measures.

**Results:**

This study will provide evidence-based guidance for the treatment of PLC with acupuncture and the external application of traditional Chinese medicine and offers new ideas and methods for the treatment of PLC.

## 1 Introduction

Primary liver cancer (PLC) is one of the most common malignant tumors in the world. Its incidence and lethality rank 6th and 3rd, respectively, among malignant tumors in the world. There were between 830,000 and 906,000 new cases of PLC diagnosed in 2020. Between 4.7% and 8.3% of new cases worldwide result in death, and this number is increasing year by year, seriously endangering people's lives and health.^[1,2]^ Although various methods such as hepatectomy, liver transplantation, local ablation therapy, and systemic therapy are currently available to treat PLC, their use is often limited by factors such as the size of a patient's tumor, a patient's liver function score, or a patient's general state of health. Therefore, the mortality rate, recurrence rate, and overall prognosis of PLC have not improved.^[3,4]^ In short, the health impacts and economic burdens of treating PLC are common problems facing the world population.^[5]^

In recent years, acupuncture combined with the external application of traditional Chinese medicine in the treatment of PLC has been recognized by doctors and patients due to its simplicity and convenience.^[6]^ As an important part of traditional Chinese medicine, acupuncture has been recognized for its effects in improving physical fitness and relieving patients’ pain. It has also been used in the treatment and care of PLC and its complications.^[7,8]^ The external application of traditional Chinese medicine as an adjuvant treatment is characterized by its low cost and direct, topical administration method, bypassing the gastrointestinal tract. These are its obvious advantages.^[9,10]^ However, the effectiveness of acupuncture combined with the external application of traditional Chinese medicine in the treatment of PLC lacks evidence-based support, and the incidence of toxicity and side effects still needs to be further verified; these are both important barriers to its application. Therefore, this systematic review and meta-analysis evaluated the efficacy and safety of acupuncture combined with the external application of traditional Chinese medicine in the treatment of PLC.

## 2 Materials and methods

### 2.1 Information sources and search strategy

This retrospective study and meta-analysis were conducted based on the guidelines of the first choice report project of the Preferred Reporting Items for Systematic reviews and Meta-Analyses (PRISMA-P).^[11]^ As this study comprised a summary analysis of data from published studies, ethical approval was not required. As of September 15, 2021, the PubMed, Web of Science, GCBI, Embase, OVID, AMED, Cochrane Library, CNKI, VIP, CBM, and Wanfang databases were searched. As of September 15, 2021, there were no restrictions on the search language, publication time, or publication status. We used the following medical keywords in our searches: “acupuncture”, “external application of traditional Chinese medicine”, and “primary liver cancer”. We also reviewed other ongoing and unpublished studies in the clinical trial registry and manually searched all reference lists from relevant systematic reviews to find other eligible studies. The expected registration has been approved by the International Platform of Registered Systematic Review and Meta-analysis Protocols (https://inplasy.com/inplasy-2021-10-0042/). And the registration number is INPLASY2021100042. The search strategy for the PubMed is presented in Table 1.

**Table 1:** The search strategy for the PubMed.

Number	Terms
#1	Primary liver cancer (all field)
#2	Hepatocellular carcinoma (all field)
#3	Intrahepatic cholangiocarcinoma (all field)
#4	Hepatocellular carcinoma - mixed intrahepatic cholangiocarcinoma (all field)
#5	Liver cancer (all field)
#6	#1 or #2–5
#7	Acupuncture (all field)
#8	Needling (all field)
#9	Acupoint (all field)
#10	Acupuncture treatment (all field)
#11	Scalp acupuncture (all field)
#12	Electro acupuncture (all field)
#13	Ear acupuncture (all field)
#14	Intradermal needling (all field)
#15	Auricular acupuncture (all field)
#16	Fire needling (all field)
#17	Catgut embedding (all field)
#18	#7 or #8-17
#19	External application of Traditional Chinese medicine (all field)
#20	External application of Chinese medicine (all field)
#21	External application of Chinese herb medicine (all field)
#22	External therapy of Chinese medicine (all field)
#23	External application of plaster (all field)
#24	Chinese medicine fumigation (all field)
#25	#19 or #20-24
#26	randomized controlled trial (all field)
#27	randomly (all field)
#28	controlled clinical trial (all field)
#29	randomized (all field)
#30	random allocation (all field)
#31	supportive treatmen (all field)
#32	single-blind method (all field)
#33	double-blind method (all field)
#34	trials (all field)
#35	comparators
#36	allocation
#37	#26 OR #27-36
#38	#6 And #18 And #25 And #37

### 2.2 Inclusion and exclusion criteria

The inclusion criteria were as follows: the study was a randomized controlled study; the included patients had PLC; the experimental group received a combination of acupuncture and the external application of traditional Chinese medicine, and the control group received the best supportive treatment; and the patient's survival, short-term curative effect and efficiency, improvement in quality of life, and occurrence of adverse events were assessed. The exclusion criteria were as follows: metastatic liver cancer; the experimental group was given other treatments; the control group did not receive the best supportive treatment but received transcatheter arterial chemoembolization, radiofrequency ablation, etc, instead; and the publication was not a research study.

### 2.3 Study selection

**Figure 1:**
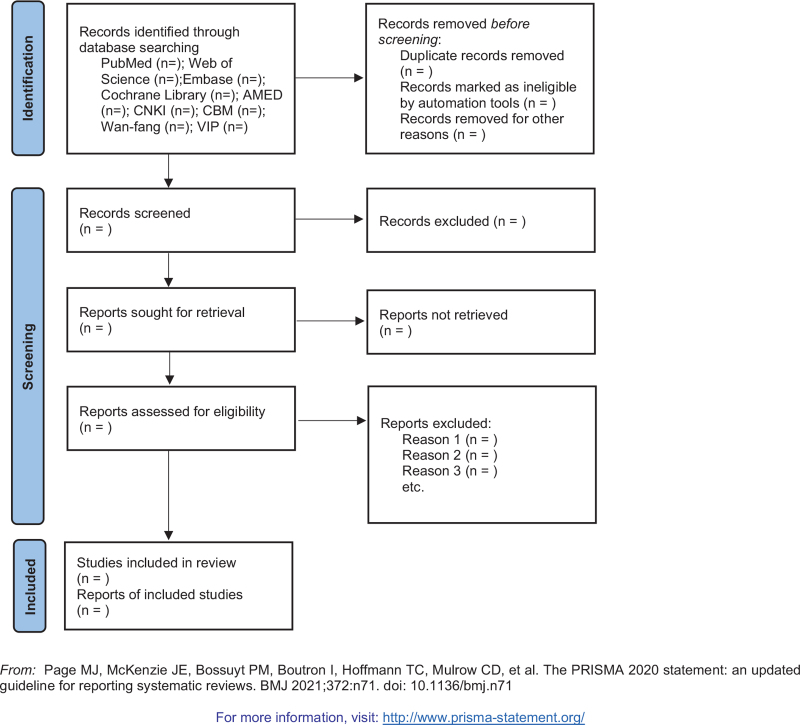
Flow diagram of study selection process.

First, 2 researchers independently screened and sorted the preliminary data according to the abovementioned inclusion criteria. Then, 2 other reviewers reviewed and re-evaluated whether the included data met the selection requirements. We will exclude all conference records, reviews, meta-analyses, newspapers, guides, letters, and other documents. During the research period, any differences between the researchers’ assessments were resolved through research and negotiation with another principal researcher until a consensus was reached. Finally, another researcher resolved any remaining differences, and then a full-text report was obtained. The research selection process is represented as a PRISMA flowchart.^[12]^ When the full text of a study or the required information in the analysis was missing, the author of the study was contacted, and the data were requested. The 2 authors independently performed data extraction according to the Cochrane manual guidelines and reported the results according to the PRISMA guidelines.^[13,14]^ Any differences were resolved by a consensus of all authors. A flowchart of the screening process is presented in Figure 1.

### 2.4 Assessment of study quality

The 2 authors separately used the Cochrane risk bias assessment tool to assess the quality of the randomized studies.^[15]^ The Cochrane bias risk assessment tool evaluates risk of bias in 6 domains: selection bias (random sequence generation, allocation hiding), implementation bias, measurement bias, follow-up bias, reporting bias, and other biases. Each item uses low bias and uncertain bias risk. In addition, the high offset is judged and divided. We used Begg test and set *P* < .1 as statistically significant, and we used a funnel chart to assess publication bias. When the quality of a single study was evaluated differently by the 2 authors, the inconsistency was resolved through a consensus among all authors.

### 2.5 Outcome measures

#### 2.5.1 Main outcomes

The short-term curative effect was measurable, patient quality of life was improved, and the survival rate of patients was between half a year and 1 year.

#### 2.5.2 Additional outcomes

Additional outcomes included the occurrence of adverse events, such as side effects, nausea and vomiting, skin allergies, and liver damage.

### 2.6 Statistical analysis

We used the random effects model in Review Manager software (REVMAN v5.3 Cochrane Collaboration) for the meta-analysis. *P* < .05 was considered statistically significant. Two authors independently performed data extraction and data input, and the third author checked the data; the first 2 authors performed the data calculations. The hazard ratio of the 95% confidence interval, or 95% CI, of the binary classification results or the continuous results was evaluated. We used I^2^ statistics to detect clinical heterogeneity, which was scored as follows: 0% ≤ I^2^ < 25%, no heterogeneity; 25% ≤ I^2^ < 50%, mild heterogeneity; 50% ≤ I^2^ < 75%, moderate heterogeneity; and I^2^ ≥ 75%, severe heterogeneity. If there was a high degree of heterogeneity between trials (I^2^ ≥ 50% or *P* < .1), we tried to determine the source of the heterogeneity through subgroup analysis, meta-regression and sensitivity analysis. Sensitivity analysis was performed by omitting individual studies. We will use subgroup analysis based on different interventions, controls, and outcomes.

## 3 Discussion

PLC is one of the most common cancers in the world. Its onset is insidious. It can be asymptomatic in its early stage. It is difficult to diagnose. When it is discovered, most patients have middle- or advanced-stage disease, which is difficult to treat and seriously endangers their lives and health.^[1–3]^ In the treatment of PLC, the external application of traditional Chinese medicine can relieve symptoms and improve patient quality of life. External treatment methods can also reduce the gastrointestinal burden caused by oral medication. External treatment is also inexpensive, simple, and convenient, and its application potential has attracted widespread attention.^[6]^ This study provides evidence-based guidance for the treatment of PLC with acupuncture and the external application of traditional Chinese medicine and offers new ideas and methods for the treatment of PLC.

## Acknowledgments

All the authors of this manuscript are very grateful to the various departments of Changchun University of Chinese Medicine for their support.

## Author contributions

**Conceptualization:** Song Wang, Tiejun Liu.

**Data curation:** Zhuang Xiong, Yangyang Liu, Yan Leng.

**Final approval of manuscript:** All authors.

**Formal analysis:** Zhuang Xiong, Dong Shen, Xiangtong Meng.

**Funding acquisition:** Zhuang Xiong.

**Investigation:** Song Wang, Zhuang Xiong, Dong Shen, Xiangtong Meng.

**Methodology:** Song Wang, Zhuang Xiong, Dong Shen, Xiangtong Meng.

**Project administration:** Yan Leng, Tiejun Liu.

**Software:** Song Wang, Zhuang Xiong.

**Supervision:** Yan Leng, Houbo Deng, Tiejun Liu.

**Validation:** Yan Leng, Houbo Deng.

**Visualization:** Song Wang, Dong Shen, Xiangtong Meng.

**Writing – original draft:** Song Wang, Zhuang Xiong, Yangyang Liu, Yan Leng, Houbo Deng, Dong Shen, Xiangtong Meng.

**Writing – review & editing:** Song Wang, Zhuang Xiong, Yangyang Liu, Yan Leng, Houbo Deng, Dong Shen, Xiangtong Meng.
